# 

**Published:** 1891-01-17

**Authors:** 


### Books Received.

Babyhood Publishing Company.
"Babyhood," volume VI.

Bailliere, Tindall, and Co.
"Aids to Sanitary Science, for the Use of Candidates for Public Health Qualifications." Francis J. Allan, M.D. Price 4s. 6d.
"Researches on Micro-Organisms." A. B. Griffiths, Ph.D., F.R.S.E., &c.

Churchill, J. and A.
"A Dictionary of Practical Medicine," by various writers. Edited by J. K. Fowler, M.A., M.D. Price 21s.
"Handbook to Dr. Koch's Treatment in Tubercular Disease." Edward F. Grun, M.R.C.S., and Walter Severn.

Hazell, Watson, and Viney (Limited).

"Hazell's Annual for 1891." Edited by E. D. Price, F.G.S.

Letts' Diaries Company, Limited (Publishers, Messrs. Cassell and Co.).
"Letts' Medical Diary for 1891."

Livingstone, E. and S. (Edinburgh).
"Manual of Surgical Anatomy." Alfred W. Hughes.

Longmans, Green, and Co.
"Evidences of the Communicability of Consumption." G. A. Heron, M.D. Price 7s. 6d.

Pitman, Frederick.

"Hygienic Medicine." J. R. Allinson, L.R.C.P. Price 1s.

Sampson Low, Marston, and Co.
"The Care of the Sick at Home and in the Hospital: A Handbook for Families and for Nurses." By Dr. Th. Billroth. Translated by J. Bentall Endean.

Unwin T. Fisher.
"Teneriffe: Personal Experiences of the Island as a Health Resort." G. W. Strettell.

Warne, F., and Co.
"Barker's Facts and Figures for 1891." Price 1s.

### NOTICE TO CORRESPONDENTS.

All MS., Letters, Books for Review, and other matters intended for the Editor should be addressed THE EDITOR, THE LODGE, PORCHESTER SQUARE, LONDON, W.

The Editor cannot undertake to return rejected M.S., even when accompanied by stamped directed envelope.

### Notice to Advertisers and Subscribers.

All Advertisements, Orders for Copies of THE HOSPITAL, and Business Communications should be addressed to The Publisher, *not to the Editor*, THE HOSPITAL, 140, Strand, W.C.

The Cost of Subscription, post free, is as follows:—Per week, 1½d.; per quarter, 1s. 8d.; per half-year, 3s. 3d.; per annum, 6s. 6d. Foreign:—Per annum, 8s. 8d.; payable, in all cases, in advance.

### Bound Volumes of "The Hospital."

Vol. VI., containing full page portraits of T.R.H. the Prince and Princess of Wales, and Miss Florence Nightingale, is still obtainable.

Vol. VII. and all the earlier volumes are also for sale.

Orders will be received for Vol. VIII., 4s. post free. Cases for binding may be had of the Publisher, at 1s. 6d. each.

### To Residents, Secretaries, and Matrons.

The Editor will be glad to have the Regulations and each Report as issued by Asylums, Hospitals, Convalescent and Nursing Institutions, as well as those of other Charities, and an early copy in duplicate of all Appeals and Festival Notices, etc., addressed to *The Editor, The Lodge, Porchester Square, London, W.* Any superintendent, secretary, matron, or other chief official who regularly sends such information may be registered, on application to the Publisher, to receive free of cost a cover for binding THE HOSPITAL in half-yearly volumes.

### BURDETT'S HOSPITAL ANNUAL FOR 1890

May be obtained of the Publisher, THE HOSPITAL Offices, 140, Strand, W.C., price 2s. 6d. limp boards, or 3s. 6d. cloth. All orders must be accompanied by a remittance.

### SCRAPS AND GLEANINGS.

The Irish Distress Fund amounted on Tuesday to £20,000.

The remains of the late Mr. A. W. Kinglake have been cremated at Woking.

Funds are much needed to provide a bath-room at the Caldicote House Convalescent Home.

The Queen has sent a present of game for the use of the patients at St. George's Hospital.

At the Hospital Morrnil in Paris, three old women were suffocated by the fumes of a defective stove.

Twenty thousand persons have died from small-pox during the recent epidemic of the disease at Guatemala.

A man has died in Sunderland from blood poisoning, caused by a nail in his boot, which punctured his foot.

Mr. G. E. W. Turner, surgeon, has resigned the offices held by him in connection with the Stockport Union.

A New Year's entertainment has been given to the in-patients of St. John's Hospital for Diseases of the Skin.

Dr. Barnardo appeals to the public for funds to carry on his work of rescue among the waifs and strays of London.

A Sicilian shoe-black, on being informed that he had won a prize of 4,000 lire (£160) in a lottery, fell dead on the spot.

Dr. Koch arrived in England on January 5th, and proceeded at once to Edinburgh. His business is of a private nature.

A man and a woman in Minnesota, U.S.A., have been married for 80 years. The man is 103, and his wife seven years older.

The Christian Community, an unsectarian benevolent society, have given food and shelter to 28,200 men during the past six years.

The death, after a brief illness, of Dr. Owen Thomas, is announced. The deceased practised surgery in Liverpool for over thirty years.

A lady, who was taken to the Stockport Infirmary to undergo an operation, died after inhaling three-quarters of an ounce of ether.

The little child David Danker, aged three years, who was terribly burned at a fire at No. 16, Samuel street, died on Thursday in the London Hospital.

The partnership hitherto existing between Mr. J. G. F. Richardson and Mr. T. H. Lloyd, under the firm of John Richardson and Co., has been dissolved.

A woman is said to be living in Vienna who has attained the great age of 116. She is still active, and sweeps and cleans up her own house, and is able to converse with visitors.

Terrible distress prevails in the Midlands. Thousands of poor people are without fires, without credit, and without food. The distress becomes more accentuated every day.

The committee of French medical men appointed to study Dr. Koch's discovery, report that it would be perhaps better to wait until Dr. Koch has perfected his method before generally adopting it.

Mr. Edward Bellamy, F.R.C.S., Fellow of King's College, London, and surgeon and lecturer on surgery at Charing Cross Hospital, died on the 4th inst, of acute pneumonia, after an illness of only three days.

The reports of the sanitary authorities at Florence state that 886 cases of typhoid fever occurred between December 15th and January 5th, with 75 deaths. The epidemic is increasing; from 100 to 150 new cases are reported daily.

The Sanitary Council of Paris, in a new code of instructions, to be hung up in the dispensaries and police-stations, prescribes vaccination as the only sure preventative of small-pox, and recommends the transport of patients to small-pox camps or hospitals.

On Wednesday an entertainment was given to the patients in the Cancer Hospital, Brompton, by Miss Rosa Leo, who was assisted by Mrs. Bunten, Miss Muriel Dowie, Mr. Bernard Lane, Mr. Frank Boor, Mr. Charles Capper, Miss Alice Bateman, Mrs. Arthur Levy, and Mr. Lander.

At an examination for inspectors of nuisances, held in London on December 4th and 5th, ninety-five candidates presented themselves, of whom fifty-one were certified to be competent, as regards their sanitary knowledge to discharge the duties as inspector of nuisances.

At St. Abbot's Hospital, Guilford, an old woman was found dead in her room. She was an invalid, and had been left but a few minutes by the nurse, when she was found lying in flames, having fallen against the fire. She appears to have died immediately. The body was terribly burned.

Drs. Behring and Kitasato believe they have found a remedy for diphtheria and tetanus. Trichloride of iodine injected under the skins of animals which have been inoculated with the virus of these two diseases, not only checks their progress, but renders the animals proof against subsequent infection.

A painful incident has occurred in a Vienna hospital. A man who is lying in the Rudolph Hospital in a dying condition, in the middle of the night stole a diamond ring from the body of a patient who had breathed his last an hour before, with his lips fervently kissing the relic of better times. The thief offered the ring to another patient for five florins, and it was thus the theft was discovered.

At the Royal Hospital for Diseases of the Chest, two wards in the new wing are entirely closed for want of funds. The annual subscriptions of this charity do not amount to £1,500, whereas the expenditure exceeds £5,000, leaving a deficit of £4,000 which has to be found each year to enable the hospital to carry on its work. The amount required to place the finances of the hospital in a satisfactory condition and to make the opening of the two empty wards possible, is about £5,000, and towards this sum the public are very earnestly asked to contribute.

The Middlesex Hospital is in want of funds. The total income for last year was £19,776, whilst the necessary expenditure was £28,590, leaving a deficiency of £8,814, to be made up out of the capital. The special feature of this hospital is the accommodation for patients suffering from cancer. There are eight beds for males and twenty-six for females. This charity is distinct, and believed to be unique throughout the world, and the maintenance of these wards, partially endowed in 1792, entails a very heavy expenditure and strain of the general funds of the hospital.

### The Student of Medicine.

[All communications for this Supplement should be addressed to The Editor of The Hospital, at 140, Strand, with the words, "Students' Column," written in the left hand top corner of the envelope.]

### EDITORIAL NOTES.

Physical Tests in Competitive Examinations.—Mr. Francis Galton has again brought before the public the scheme which he first proposed at the meeting of the British Association two years ago. The general idea is, that in public examinations the candidates should receive marks not only for the set papers, but also according to their individual physical efficiency. That literary examinations are not, as has been stated, indirect tests of bodily efficiency has been shown. There has been a vast amount of lax assertion in reference to this matter, some having said that high intellect is often associated with a stunted and weakly frame, and others have pointed to instances in which high mental and high physical powers were connected. Dr. Venn has discussed the various measurements of Cambridge students as follows: The number of those who were measured was 1,905, and they were divided into three classes—(1) high honour men; (2) low honour men; and (3) poll men. The result was that the physical efficiency of the three classes proved to be almost exactly the same, except that there appeared to be a slight deficiency in eyesight among the high honour men. Mr. Galton says, "Therefore the fact that a man had succeeded in a literary examination does not give the slightest clue to the character of his physical powers." The intellectual differences are usually small between the candidates who are, according to the present literary examinations, placed near to the dividing line between success and failure. But their physical differences may be very great. Mr. Galton holds that when two candidates are almost on a par intellectually, and one is far superior physically, that the latter should be preferred. This scheme certainly has much to recommend it, and would be very welcome to all true athletes; many would be delighted to hear that at a critical point in their examination the result, either of six months or a pass, depended upon them passing satisfactorily one or more of Galton's tests, such as stature, span of arms, weight, strength of grasp, breathing capacity, quickness of response to a signal by sound, quickness of muscular action, tests of vision, or a test of hearing powers.

Undergraduates and Funerals.—There was recently a paragraph in some of the daily papers on the reported determination of a certain Scottish professor not to permit the students attending his lectures to be absent for more than one family funeral during each session. According to the professor's account, there has been of late a most prodigious mortality among aunts and other relatives of the undergraduates. The interesting morbid desire to wander in church-yards and be present at various not over-pleasing formalities does not, as a rule, affect the student as much as certain types of people of more advanced years. We are disposed to look upon the story as a joke on the part of some of the students rather than as a serious law laid down by the professor. The story, however, reminds us of the case of the undergraduate at Cambridge applying to his tutor for an exeat to attend his mother's funeral, the tutor saying he would give him permission this time, but it must not happen again.

### LEADING ATHLETES.

Mr. Mitchell, the well-known football player, started the game at a very early age while in Canada. He learnt the Rugby rules at Browsgrove School, the head-master of which, Mr. H. Milkington, being one of the best judges and keenest supporters of the game in the midland counties. After leaving school he entered Caius College, Cambridge, in 1884. During the first season he was only able to play in a few matches owing to synovitis in the knee-joint. The next season, 1885-6, he played in the Caius Rugby team, and also played at three-quarter back for the University team some half-a-dozen times before the Oxford and Cambridge match, and regularly for the rest of the season. Next season, 1886-7, he was again prevented from playing, on account of his injured knee, till about four matches before the Inter-'Varsity match, when he obtained his much coveted "blue" as back, only having played back in two previous matches out of the four. Since this time he has played regularly for Richmond, when not playing for Guy's Hospital, which he entered after his University career. In 1887-88 he played three-quarter back for London versus the Western Counties. In 1888-89 he played in all the representative matches, including the two North versus South's, as full back. In 1889-90 he played in some representative matches, as in the previous year, and in the International team against Scotland, Ireland, Wales, and also for England against Yorkshire. In addition to these Rugby games, he has also played for Caius and Guy's in their Association Cup ties. Not only has Mr. Mitchell obtained this renown in the football world, but he has also represented both his college and his hospital at cricket, and has been in three winning teams in the Cup ties out of his four years at Guy's. At Cambridge he also obtained the freshmen's long jump and weight in 1885, and represented the Varsity in putting the weight as second strong in 1887, and obtained the hurdle race and hammer in the United Hospitals sports in the same year.

LEADING ATHLETES.

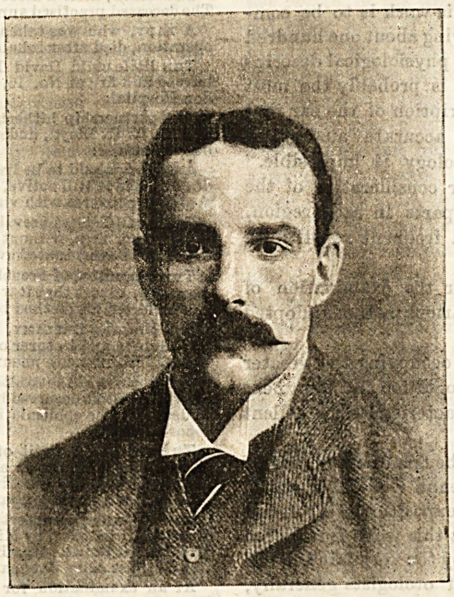

*From a photograph by Walery.*

Mr. W. G. MITCHELL (Guy's).

### NOTES FROM THE SCHOOLS.

### CHARING CROSS HOSPITAL.

The Charing Cross Hospital Students' Club gave their twelfth annual entertainment to the patients and visitors in the Board Room of the Hospital on Wednesday, December 17th. The performance commenced at 6.45 with an overture, followed by the farce "Chiselling," in one act, in which Miss Ethel Pink, and Messrs. Whitehead, Corbould, Gordon, and P. Forbes-Winslow took part. After an interval, during which the visitors inspected the wards of the hospital, songs were sung by Mr. A. E. Reade and Miss Kempster, followed by a valse by the orchestra. The last item on the programme was the well-known comedy "Naval Engagements," in which Miss Ethel Pink, Miss Birch, and Messrs. Harold Gardner, P. Forbes-Winslow, Ellis Clowes, and H. Whitley took part. The twelth annual entertainment of the Students' Club was voted a great success by all who were fortunate enough to have been present, and showed considerable musical and dramatic talent in some of its members.

### UNIVERSITY COLLEGE.

Mr. W. F. R. Weldon, M.A., F.R.S., Fellow of St. John's College, Cambridge, has been appointedby the Council of University College, London, to the Jodrell Professorship of Comparative Anatomy and Zoology, which was held for sixteen years by Professor Ray Lankester. Mr. Weldon at present is a lecturer on Invertebrate Morphology to the University of Cambridge. Mr. F. E. Weiss, B.Sc., has been elected to the Quain Studentship in Botany; and Mr. T. W. P. Lawrence, M.B., F.R.C.S., has been appointed Curator of the Anatomical Museum.

### CAMBRIDGE UNIVERSITY.

The following degrees were conferred on December 20th: Doctor of Medicine, Lewis Erle Shore, St. John's; Doctor in Science, Micaiah John Muller Hill, Peterhouse.

The Hutchinson studentship at St. John's College, of the value of £60, open to students of the college engaged in physical or natural science studies, who must be of not less than nine and not more than 18 terms' standing, has been awarded to John Theodore Hewitt, B.A., scholar of the College, for chemistry. Mr. Hewitt obtained a first class in the Natural Sciences Tripos, Part I., June, 1889, and was placed in the first class Natural Sciences Tripos, Part II., June, 1890, for proficiency in chemistry.

Entrance Scholarships in Natural Science.—Christ's College, A. W. Rogers, Clifton College, £60; E. B. H. Panton, Dulwich School, £40; A. E. Jeaffreson, St. Paul's School, £30.

The Bachelor Scholarship of £50 has been awarded to A. H. L. Newstead, for Zoology. At Emmanuel College H. M. King, Dulwich College, £40 exhibition; A. E. Porter, Clifton College, £30 exhibition.

### APPOINTMENTS.

At Guy's Hospital, F. W. Hall, M.B., B.S.Lond., has been appointed house physician; H. M. Jordan, M.R.C.S. L.R.C.P., has been appointed house surgeon; H. A. Smith, M.B., B.C. Camb., has been appointed house physician; R. G. P. Lansdown, M.B., B.S. Durh., has been appointed house surgeon; H. W. Webber, M.B., B.S.Lond., has been appointed assistant house physician. At Charing Cross Hospital, Geo. F. Dickinson, L.R.C.P.Lond., M.R.C.S., has been appointed house surgeon; John Harold, L.R.C.P.Lond., M.R.C.S., has been appointed house physician; D. J. Jones, L.R.C.P. Lond., M.R.C.S., has been appointed resident obstetrical officer. At the Royal Infirmary, Newcastle-on-Tyne, Henry Brunton Angus, M.B., B.S., has been appointed house surgeon. At the North-Western Fever Hospital of the Metropolitan Asylums Board, William Henvey, M.R.C.S., L.R.C.P.Lond., has been appointed second assistant medical officer. At the Victoria Hospital for Sick Children, Queen's Road, Chelsea, Arthur Gale, M.R.C.S., L.R.C.P., has been appointed house physician, Robert Nairn, M.D., L.R.C.P., has been appointed house surgeon. At the West London Hospital, Hammersmith Road, F. H. Alderson, M.B.Durh., L.R.C.P. Lond., M.R.C.S.Eng., has been appointed house physician. At the Sheffield Public Hospital and Dispensary, F. J. Green, B.A., M.B., B.Ch.Dub., has been appointed assistant house surgeon; J. Jeeves, L.R.C.P.Lond., M.R.C.S.Eng., has been appointed house surgeon. At the General Hospital for Sick Children, Pendlebury, Manchester, F. H. Westmacott, M.R.C.S., L.R.C.P., has been appointed junior resident medical officer. At Alnwick Infirmary, Benjamin Sweeten, M.B., C.M.Edin., has been appointed house surgeon. At University College Hospital, Lewis Williams, M.B., B.S.Lond., M.R.C.S., L.R.C.P., has been appointed house physician. At Charing Cross Hospital, D. J. Jones, L.R.C.P. Lond., M.R.C.S.Eng., has been appointed resident obstetrical officer. At the Glasgow Eye Infirmary, Leslie Buchanan, M.B., C.M.Glasgow, has been appointed house surgeon. At the London Hospital, Henry Percy Dean, M.S., M.B., B.Sc.Lond., F.R.C.S.Eng., has been appointed surgical registrar. At the Richmond Asylum, Dublin, J. N. Eustace, M.B., B.Ch.Dub., has been appointed resident assistant clinical clerk. At the Children's Hospital, Birmingham, William J. L. Ffrench, L.R.C.P., F.R.C.S.Ireland, has been appointed assistant resident medical officer. At the Hertford British Hospital, Paris, Hugh Highet, M.B., C.M.Glas., has been appointed house surgeon. At the Royal Albert Hospital, Devonport, George G. Parsons, L.R.C.P.Lond., M.R.C.S., has been appointed assistant house surgeon. At the Hospital, Barrow-in-Furness, John B. Vickers, L.R.C.P.Lond., M.R.C.S., has been appointed house surgeon. At the Westminster Hospital, G. B. James, M.R.C.S., L.R.C.P., has been appointed senior house surgeon; Eric H. Sharman, M.R.C.S., L.R.C.P., house surgeon; and A. M. Gossage, B.A. Oxon., M.R.C.S., L.R.C.P., resident obstetric assistant. At the Monsall Fever Hospital, Manchester, George Ernest Fryer, L.R.C.P.Lond., M.R.C.S.Eng., has been appointed assistant medical officer. At the Mansfield Woodhouse Hospital, G. P. Godfrey, L.R.C.P., L.R.C.S.Edin., has been appointed visiting house surgeon. At the Royal Infirmary, Manchester, Otto Jackson Kauffmann, M.D.Lond., M.R.C.P., has been appointed resident medical officer. At the Devon and Exeter Hospital, Mr. Martyn has been appointed house surgeon. At the British Hospital, Buenos Ayres, John O'Conor, M.A., M.D., B.Ch. Dub.Univ., has been appointed resident medical officer. At the Royal Free Hospital, Gray's Inn Road, M. A. Trevithick, M.B., B.C.Cantab., M.R.C.S.Eng., has been appointed junior resident medical officer. At the Morpeth Dispensary, Stanley Yeoman, B.A., M.B., B.C.Cantab., M.R.C.S.Eng., has been appointed house surgeon. At the Essex and Colchester Hospital, W. F. A. Clowes, M.R.C.S., L.R.C.P., has been appointed house surgeon.

### STUDENTS' MEDICAL SOCIETIES.

#### Guy's Hospital Pupils' Physical Society.

A meeting of this society was held on Saturday, January 10th, when there was a clinical evening. The chair was taken by Mr. H. V. Hickman, and the following gentlemen showed cases, which were afterwards discussed. Mr. H. M. Jordan showed a boy with lupus of the nose and buttock, treated by Koch's injection. Mr. Guy Mackeson showed a boy with general lupus of

### The Hospital Advertiser.

### Students' Medical Societies—(*continued*).

the trunk and one arm, and a girl with lupus of the nose, both of whom had been injected with Koch's fluid. In every case febrile reaction had been followed by considerable improvement of the diseased patches. Mr. C. Spurrell showed a woman with an obscure swelling in the interscapular region. The only suggestion as to diagnosis was ossification in the rhomboid muscles. Mr. A. J. Sharp showed a man with involuntary athetotic movements of the fingers and toes of one side, due to an injury thirty-five years ago. Mr. Gregar showed a man with an abscess, presumably tubercular, pointing at the anterior chest wall. Mr. G. T. Birdwood showed a case of locomotor ataxy with diplopia, Argyll-Robertson pupil, analgesia, and loss of knee-jerks, but no anæsthesia or lightning pains. No history of syphilis could be ascertained. Mr. J. M. Gill showed an old dislocation of the shoulder, which had been converted by manipulation from subclavicular to subcoracoid. Excision of the head of the humerus was the treatment proposed. The next meeting will be on Saturday, January 24th, at half-past seven p.m., when Mr. A. M. Daldy will read a paper on "Cold, and the Disorders Caused by it; and Ice in the Treatment of Disease."

### PRESENTATION TO A MEDICAL STUDENT.

A few days ago the citizens of Bedlington, Northumberland, entertained Mr. Cuthbert Rutherford, a student at the Royal Infirmary Medical School, Glasgow, at a banquet. Mr. James Trotter was in the chair. After the dinner Mr. Rutherford was presented with several cases of surgical instruments, as a token of the agility he had displayed on behalf of many public events connected with the neighbourhood. In making the presentation, the chairman said that they could not but highly appreciate the keen interest taken by Mr. Rutherford in the education of the people in scientific truth, and admire the uncompromising opposition he offered to complacent orthodoxy wherever it was to be found.

### THE STUDENTS' BOOKSHELF.

We omitted to state with reference to the "Manual of Urine Testing," noticed in our number of December 27th, that the publishers were Messrs. Mullan and Son, Belfast. The book may be obtained from Mr. John Scott, Windsor Park, Belfast.

### SCHOLARSHIPS AND PRIZES.

### Owens College, Manchester.

The following elections to Fellowships have recently been made by the council for the year 1891: Bishop Berkeley Fellowships, Walter Garstang, M.A., in Zoology; W. R. Ormandy, in Chemistry; and renewed for a second year to P. J. Hartog, B.Sc., in Chemical Physics; H. W. Pomfret, M.D., F.R C.S., in Pharmacology. Honorary Research Fellowships, E. J. Bles, B.Sc , in Zoology; Gibson Dyson, Ph D., in Chemistry; J. L. Hoskyns-Abrahall, B.A., Ph.D., in Chemistry; C. H. Lees, M Sc., in Physics.

### Cambridge, Jan. 5.

At Sidney Sussex College the following have been recommended for election to scholarships and exhibitions open to persons not yet in residence: Natural Sciences Scholarship, £60, A. C. Jordan, Manchester Grammar School; exhibitions, £30, F. G. Thomas, Exeter School; E. M. Corner, Epsom College.

### PASS LISTS.

### London, Jan. 5.

EXAMINATION FOR HONOURS. (B.A. AND B.Sc. CONJOINTLY.)

MATHEMATICS.—*First Class*: Crossley W. C. Barlow, B.A. (disqualified by age for the scholarship), St. Peter's Coll., Camb., and Gilbert T. Walker, B.Sc. (scholarship), Trinity College, Cambridge, equal; George A. Schott, B.Sc., Trinity College, Cambridge.

MENTAL AND MORAL SCIENCE.—*Second Class*: E. Wesley Thompson, B.A. Univ. C. Aberyst. and Kingswood S., and Emmanuel Wheeler, B.A., Mason College, equal. *Third Class*: Thomas Phillips, B.A., University College, Bangor; F. Warburton Lewis, B.A.

(B.Sc. ONLY.)

CHEMISTRY.—*First Class*: John T. Hewitt (scholarship), Hartley Inst., Norm. Sch. Sci., and St. John's College, Camb.; Duncan S. Macnair, Owens C. and Univ. Wurzburg; Frederick D. Chattaway, Un. C. Aberyst. and Ch. Ch. Oxford; John Wade, Guy's Hosp., Univ. C. *Second Class*: Frederick W. Short, Pharmaceutl. Soc and Birkbeck I.; James Hendrick, King's Coll. and Birkbeck Inst. *Third Class*: Thomas G. Nicholson, St. Thomas's Hospital; Frederick G. Hopkins, Guy's Hospital.

EXPERIMENTAL PHYSICS.—*First Class*: William Watson (scholarship), Normal Sch. of Sci.; Alfred W. Porter, Univ. Cs. L'pool and Lond.; Owen G. Jones, City Guilds Centr. Inst. *Third Class*: Harry E. Hadley, Arthur R. Hall Ingram, Mason College; Thomas P. Nunn, Univ. Coll., Bristol; George A. Schott, Trinity College, Cambridge; Walter Thorp, Yorkshire and University Colls.; John T. Hewitt, Hartley Inst., Norm. Sch. Sci., and St. John's College, Camb.

PHYSICAL GEOGRAPHY AND GEOLOGY.—*First Class*: William Fraser Hume, Normal Sch. of Sci.; Arthur Thomas Simmons, Normal Sch. of

**Pass Lists**—(*continued*).

Sci. *Second Class*: Herbert George Wells, Normal Sch. of Sci.; Sarah Beatrice Lowe, Westfield and Univ. Colleges. *Third Class*: James Alfred Butterfield; John Henry Salter, Dalton Hall and Owens College; John William Martin, Birkbk. I., Bromley S. Sc.

BOTANY.—*First Class*: Arthur H. Church, Univ. Coll., Aberystwith; *Second Class*: Claude H. C. Woodhouse, Normal School of Science. *Third Class*: J. S. Collier, St. Mary's, City Guilds; Charles F. Rea, B.A., King's College; John H. Salter, Dalton Hall and Owens College.

ZOOLOGY.—*First Class*: Ernest W. H. Groves, University Colls., Bristol and Lond., and Arthur Willey, University College (equal); Herbert G. Wells, Normal Sch. of Sci. *Second Class*: Chas. Wm. Andrews, B.A., Birkbeck Institution; Florence Buchanan, Univ. Coll. and Lond. Sch. of Med.; and John Stephenson, Owens College (three equal); John H. Salter, Dalton Hall and Owens Coll. *Third Class*: George H. Carpenter, King's C. and R. C. Sci., Dublin; Edward J. Bles, Owens College.

PHYSIOLOGY.—*First Class*: Bertram L. Abrahams (scholarship), University College; John Le Mare Bunch, University College (obtained the number of marks qualifying for the University Scholarship). *Second Class*: John H. Parsons, University Colls., Bristol and London, and William B. Winston, St. Thomas's Hospital and Birkbeck Institution (equal). *Third Class*: Frederick G. Hopkins, Guy's Hospital; John S. Sloane, St. Bartholomew's Hospital.

B.S. EXAMINATION 1890. EXAMINATION FOR HONOURS.

SURGERY.—*First Class*: E. V. Hugo (Gold Medal), St. Bartholomew's Hospital; W. H. Evans, M.B., B.Sc. University College. *Second Class*: T. J. Dyall, St. Bartholomew's Hospital; A. W. W. Lea, Owens College and Manchester Royal Infirmary; F. W. Hall, Guy's Hospital.

**Colonial Examinations, Jamaica.**

B.S. EXAMINATION. EXAMINATION FOR HONOURS.

SURGERY.—*First Class*: Edward V. Hugo (gold medal, St. Bartholomew's Hospital; W. H. Evans, M.D., B.Sc., University College *Second Class*: Thomas J. Dyall, St. Bartholomew's Hospital, and Arnold W. W. Lea, Owens College and Manchester Royal Infirmary, equal; Frederick W. Hall, Guy's Hospital.

### ATHLETICS.

### Football.

RUGBY UNION RULES.

THE RUGBY UNION AND THE LAWS OF THE GAME.—The following decisions have been given by the Rugby Union on the laws of the game:—(1) Law 43.—In case of a fair catch when the kicker retires behind his own goal line to take his kick. Decision—"The players of the kicker's side must be behind the kicker, and cannot claim to come up and stand on the goal-line under Law 21, which states 'No player can be off-side in his own goal.'" (2) A referee has no power to order a scrummage five yards back from the goal-line, but is recommended to allow it if both captains agree. (3) Law 46, which states: If a player shall kick, pass, or carry the ball back across his goal-line and it there be made dead, the opposite side may claim that the ball shall be brought back and a scrummage formed at the spot whence it was kicked, passed, or carried back. Decision—"A player who carries the ball behind his line, even if pushed by an opponent, and there touches it down, has broken the law."

LAST Saturday, namely, January 10th, was the fifth in succession, there have been no metropolitan football matches owing to the severity of the frost. Such an interruption to the game is quite a novel experience to the present generation of football players. From time to time it happens in the course of the season that for one or occasionally two weeks the fixed matches are interrupted, or postponed; but nearly six weeks without a Metropolitan Hospital match is not what our players expect. In the metropolitan area the following are among the important matches that were to have been decided on Saturday last. St. Thomas were to have met Kensington at Wood Lane, and Guy's to have gone to Croydon to have played the town club there. St. Bartholomew's Hospital had a fixture with the Middlesex Wanderers; Charing Cross Hospital were to have met Kingston at Kingston. Also the interesting return match between London Scottish and Blackheath should have been played in the Old Deer Park at Richmond, the rivalry between these two well-known clubs being very keen. This match would have created considerable attention, the former engagement at Blackheath being only won by a small point by Blackheath. Several matches were to have been played on the adjoining ground of the Richmond Athletic Company had the weather permitted. Under the Association rules the, as yet, unplayed games in the first round of the London Senior Cup were to have been played off. In addition to these St. Bartholomew's Hospital were to have played Woodville at Walthamstow. So also a match between the Casuals and Stoke should have come off at the Oval, Crusaders and Old Harrovians at Harrow, Old Carthusians versus Old Foresters at Walthamstow, Clapham Rovers and Chiswick Park, Streatham, Old Wykehamists and Casuals at Wormwood Scrubs.

JAN. 2ND.

*Edinburgh Academicals v. Melrose.*—The Academicals won easily, beating their opponents by one goal and four tries to nil.

ON Monday, the 12th, the annual match between *North and South* was played at Nottingham. The result was the Southerners lost a pluckily-fought game by three goals to none. Geary registered the first goal for the Northerners within the first fifteen minutes from the start, and Chadwick with a screw-kick obtained the second goal, the third being obtained by Townley.

**EVENTS FOR THE MONTH OF JANUARY.**

[Items intended for this column must reach the office, 140, Strand, W.C., not later than first post on Monday mornings.]

For list of Students' Medical Societies' Meetings and Football Matches during January, see THE HOSPITAL for Nov. 29th.

